# Pyrazinyl and pyridinyl bis-azomethines formation: an experimental and computational study

**DOI:** 10.1038/s41598-023-44585-7

**Published:** 2023-10-13

**Authors:** Radek Coufal, Jiří Vohlídal

**Affiliations:** 1 Department of Science and Research, Faculty of Health Studies, Technical University of Liberec, Studentská 1402/2, 461 17 Liberec 1, Czech Republic; 2 Department of Physical and Macromolecular Chemistry, Faculty of Science, Charles University, Hlavova 8/2030, 128 40 Prague 2, Czech Republic

**Keywords:** Chemistry, Organic chemistry, Physical chemistry, Theoretical chemistry

## Abstract

Formation of bis-azomethines from hydrazine and heterocyclic aromatic carbaldehydes, namely pyridine-2-carbaldehyde and pyrazine-2-carbaldehyde, is studied using density functional theory. The theoretical investigation is correlated with experimental results obtained by means of NMR spectroscopy. The presence of bis-hemiaminal intermediates is evidenced by NMR spectra while surprisingly stable hemiaminal intermediate was isolated experimentally. Water, methanol and acetic acid were outlined to play a crucial role as active catalysts of elementary steps of the reaction mechanisms. The possible reaction sequences, i.e. addition-dehydration-addition-dehydration or addition-addition-dehydration-dehydration are investigated and discussed. Also, alternative mechanistic path via ionic mechanism was proposed for the formation of hemiaminals.

## Introduction

**Figure 1:**
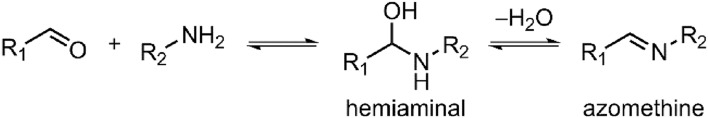
Azomethine formation.

Since the first report by Hugo Schiff in 1864^1^ imine compounds (also referred as azomethines or Schiff bases) have drawn significant attention in various fields of science. Experimentally feasible condensation reaction between an amine and carbonyl compounds to form imines (Fig. 1) is manifested by many vital applications in coordination and supramolecular chemistry^2–9^, organic electronics^10^, organic light-emitting devices (OLEDs)^11,12^ or fluorescent dyes^13,14^. Due to the reversible nature, imine formation is also at the forefront of constitutional dynamic chemistry (CDC)^15^.

It has been well established that the reaction proceeds through an intermediate tetrahedral hemiaminal, generally expected to be short lived. Accordingly, the hemiaminal usually proceeds back to starting compounds or eliminate a water molecule forming a stable imine. Due to their low stability, reports on hemiaminals are relatively scarce. However, several sophisticated methods have been explored to observe or allow the isolation of a hemiaminal. A molecular cavitands employed by Rebek et al.^16,17^ or Yang et al.^18^ and porous crystalline network by Kawamichi et al.^19^ or Yaghi et al.^20^ were utilized for kinetic stabilization of the hemiaminal. Also, solvent free conditions^21^ or targeted design of reacting components^22–24^ may enable hemiaminal observation. Hemiaminal-based system has been also implemented in CDC^25–27^ (or more specifically in dynamic covalent chemistry, DCC) and demonstrated as chiral sensing system^26,27^.

The mechanism of imine formation has been the subject of several studies^28–37^, however, understanding of elementary chemical events on such a multi-step process remains limited. Moreover, mechanistic studies dealing with high-cost energetics for the nucleophilic addition or dehydration steps have appeared in the literature^28,37^. Furthermore, the reports have been focused to a single imine bond formation. Extensive theoretical examinations of addition of ammonia to formaldehyde were performed by researchers to advocate the occurrence of the abiotic formation of α-amino acids on the early Earth through Strecker synthesis^36^. However, questions with regard to the mechanism and order of elementary steps for the formation of diimine or azine products remain open.

**Figure 2:**
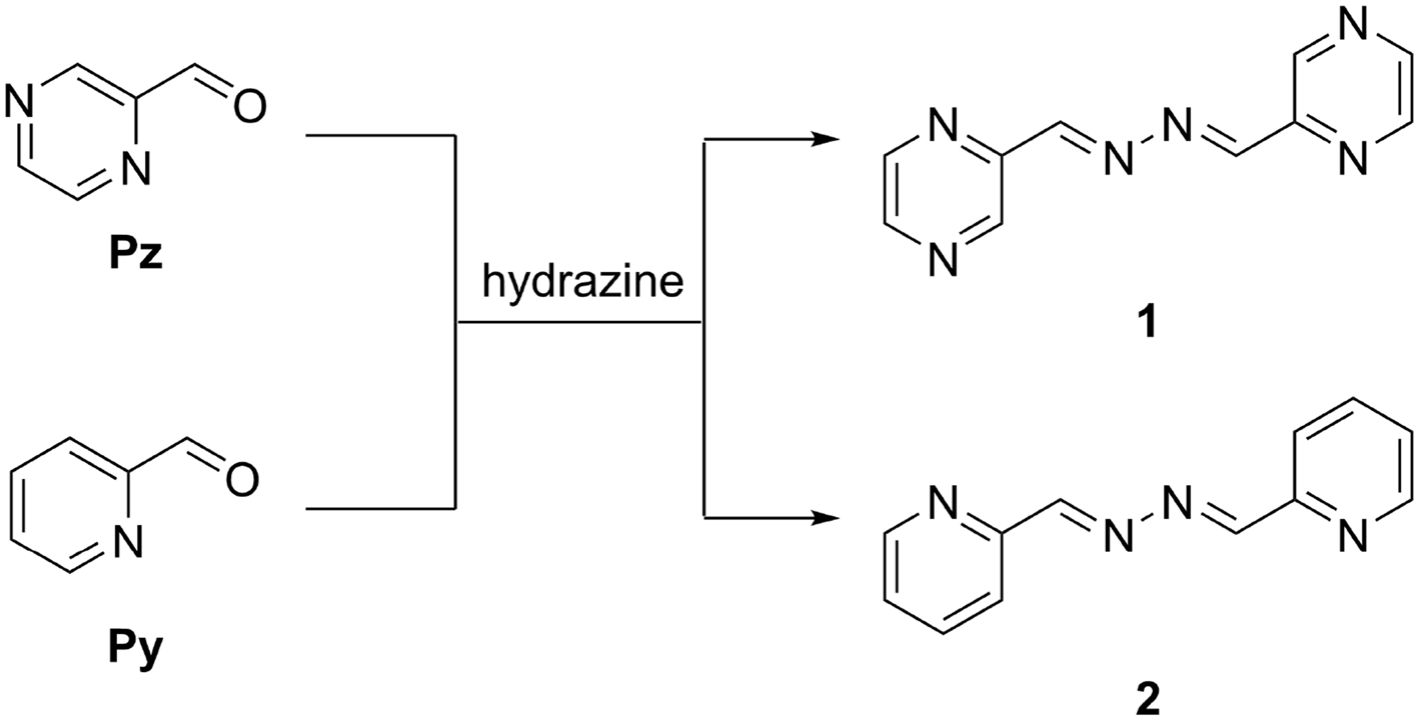
Synthesis of azines.

Herein, our aim was to synthesize and characterize azines **1** and **2** (Fig. 2). Notably, we have found during our effort that pyrazine-2-carbaldehyde forms stable hemiaminal intermediate which can be isolated. Moreover, we were able to detect open-chain bis-hemiaminal structure. These results prompted us to examine mechanistic pathways leading to azines **1** and **2** in details and we aim to shed light on the experimental results.

## Methods

### Kinetic experiments

The stock solutions for NMR kinetic measurements were prepared (just before use) by weighing (± 0.1 mg) into a 2 mL volumetric flask followed by addition of THF-d8 to form solutions of 0.25 M. Then, the tempered solution of hydrazine (0.25 mL) was added to the tempered solution of the respective carbaldehyde (0.5 mL) in NMR tube using appropriate microsyringe (molar ratio 1:2). NMR tube was kept airtight throughout spectra aquisition at 273.2 K. The kinetic build-up curves for the rate-determining steps enabled reliable fitting with a second-order polynomial function (see Fig. S1) and the first derivates at time zero are used as the rate constants (linear approximation of the initial growth). Then, the Gibbs free activation energies were obtained using transition state theory.

### Computational details

All structures employed in this study were optimized using the M06-2X functional^38^ in conjunction with the 6–311 +  + G(d,p) basis set, as implemented in the Gaussian 09 program package^39^. Frequency analyses at the same level of theory were performed in all the stationary geometries in order to assign them as genuine minima (no imaginary frequency) or transition states (TS, only one imaginary frequency corresponding to the reaction coordinate) on the potential energy surface (PES) as well as to evaluate the thermochemical corrections at 298.15 K. A pruned (99, 590) UltraFine grid was used for the numerical computation of two electron-integrals. The connectivity of each TS with the associated minima was described using IRC (intrinsic reaction coordinate) procedure^40–42^. For stationary points a detailed conformational analysis was carried out. Relaxed PES scan was performed for at least two torsion angels, starting from the optimized minima following the IRC. Angles were varied from − 180° to + 180° with step size 6° and geometrical structures with the lowest electronic energy found at PES were subsequently reoptimized. All diastereoisomers (and TS leading to them) were evaluated and only the most stable structures are given. The solvent effects were estimated through the SMD model^43^ in tetrahydrofuran or methanol as the continuum dielectric (*ε* = 7.4257 and *ε* = 32.613) within reoptimization of the located stationary points at the same level of theory. Analytical Hessians were again calculated to control the identity of the stationary points as well as to obtain the thermochemical corrections at 298.15 K. Additional single points energy calculations were conducted at M06-2X/6–311 +  + G(d,p) level of theory to obtain the solvation free energies. The final energy diagrams were obtained by calculating solution phase free energies according to Eq. (1):

1 $$G_{{{\text{soln}}}} = E_{{{\text{gas}}}} + G_{{\text{n}}} + \Delta G_{{{\text{solv}}}} + { 1}.{\text{89 kcal mol}}^{{ - {1}}} ,$$

where *G*_soln_ refers to solution phase free energy, *E*_gas_ refers to electronic energy obtained in the single point calculations at M06-2X level in conjunction with def2-TZVPPD basis set, Ref.^44^ *G*_n_ refers to thermal correction to Gibbs free energy obtained at M06-2X/6–311 +  + G(d,p) level and Δ*G*_solv_ is solvation free energy. The final term 1.89 kcal mol^−1^ converts from the gas-phase standard state (defined by *T* and *P*) to the solution-phase standard state of 1 M.

The relative Gibbs free energies of intermediates, in kJ/mol, are calculated as difference of the sum of the isolated reactants and the particular intermediate. The Gibbs free activation energies are calculated as the difference between the transition state (TS) and the respective hemiaminal (dehydration step) or as the difference between isolated reactants and the TS (the first addition step).

## Results and discussion

Azines **1** and **2** were synthesized by the standard procedures^7^. The synthesis and structure of azine **2** was reported before^45^. X-ray structure of newly prepared azine **1** is shown in the SI (Fig. S6).

Experimental and theoretical bond lengths and angles are given in Table 1. Theoretical structures of azines **1** and **2** were obtained independently by the proposed reaction mechanisms (vide infra) and satisfactory agreements with experimental structures were reached (see Fig. S5). Both azines are planar with the dihedral angles of 180.0° (both experimentally and theoretically) about the azine linkages, supporting conjugation throughout the *π* systems. The electronic communication is reflected in the N–N bond lengths with the values of 1.408 for **1** and 1.413 Ǻ for **2**, which is significantly shorter than the N–N single bond length in hydrazine (1.449 Ǻ)^45,46^. This effect may be also partially caused by conjugation of nitrogen lone pairs into the *π* systems of azines **1** and **2**, increasing the imine bond lengths. Furthermore, stronger electron-withdrawing character of the pyrazine ring with respect to the pyridine ring is manifested by the shorter N–N bond length in **1** with respect to **2**. This is further justified by the longer imine bond length in **1** with respect to **2**. The C = N–N angles of 111.7 and 111.8° in **1** and **2**, respectively, reflect the repulsion of the nitrogen lone pair and the double bond. The C–C = N angles are much closer to the sp^2^ value (120°), in **1** with the value of 120.8° being slightly closer than in the case of **2** (122.6°).

**Table 1:** Experimental and theoretical (in parentheses) bonds lengths (Å) and angles (deg).

	*l*_N–N_	*l*_C=N_	*α*_C=N–N_	*α*_C–C=N_
1	1.408 (1.390)	1.273 (1.274)	111.7 (112.2)	120.8 (120.8)
2	1.413 (1.391)	1.266 (1.274)	111.8 (112.2)	122.6 (121.1)

Structure of the azine **2** was reported in Ref.^45^.

**Scheme 1:**
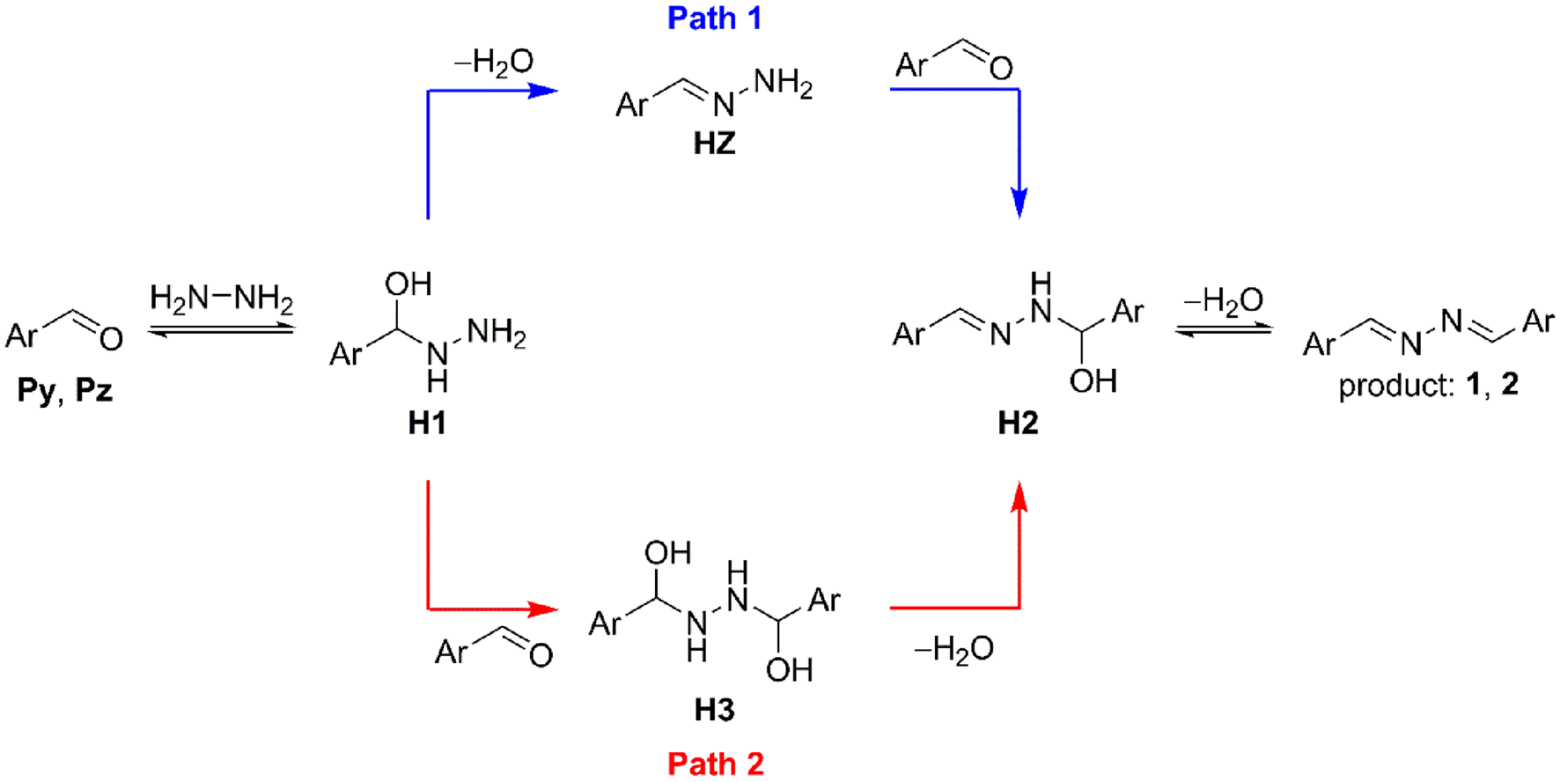
Possible mechanistic paths for azine formation. Ar stands for pyrazine or pyridine ring.

The possible reaction paths for the formation of azine **1** and **2** are depicted in Scheme 1. Evolution of intermediates in the reactions of pyridine (**Py**) and pyrazinecarbaldehyde (**Pz**) with hydrazine was monitored by ^1^H NMR. Hydrazone **HZ**, hamiaminal **H2** and bis-hemiaminal **H3** can be clearly resolved in NMR spectra (Figs. S7–S10) in the course of reactions. For hemiaminal **H2** and bis-hemiaminal **H3**, characteristic resonances for protons on carbon stereogenic centers appeared at around 6 ppm and 5.5 ppm, respectively. Further, azomethine protons of the hemiaminal **H2** which appeared around 7.6–7.8 ppm are well-separated from resonances at around 7.7 ppm which are assigned to azomethine protons of the hydrazone **HZ**. Also, resonances corresponding with exchangeable protons (NH, OH) of the hemiaminal **H2** can be observed in the spectra. Assignment of these resonances is confirmed by increasing temperature (causing downfield shift) and also by coupling interaction with C-H protons. Formation of bis-hemiaminal **H3** is clearly reflected in two short lived sets of resonances with close chemical shifts corresponding with two diastereoisomers in approximately equal proportion. The presence of **H3** is further confirmed by the resonances at around 5.4–5.5 ppm assigned to protons on carbon stereogenic centers which are shifted more downfield compared to that of the hemiaminal **H2**.

**Figure 3:**
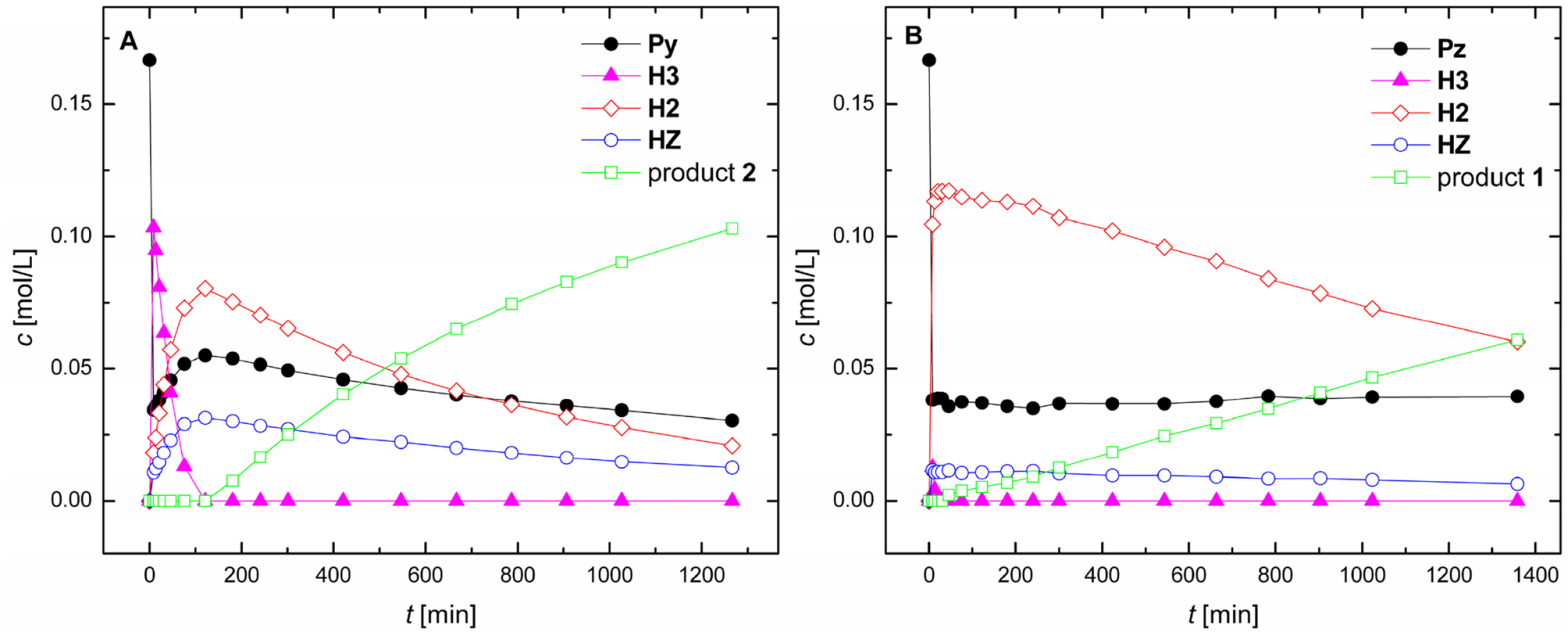
^1^H NMR kinetic profiles for reaction of pyridine-2-carbaldehyde (**A**) and pyrazine-2-carbaldehyde (**B**) with hydrazine. Reaction conditions: THF-d8, *c*(aldehyde) = 0.25 M, molar ratio 2:1, *T* = 273 K.

The kinetic profiles for the reaction of hydrazine with **Py** and **Pz** are shown in Fig. 3. In the case of **Py**, within the first minutes of mixing, the concentration of the pyridinyl bis-hemiaminal **H3** was seen to build up and decline very fast (Fig. 3A). At the same time, concentration of **Py** declines sharply first and then slightly builds up to reach approximately steady state as time progresses. We interpret these observations to correspond with the theoretically proposed Path 2 with the two consecutive low energy demanding addition steps. Considerably higher relative abundance of the hemiaminal **H2** with respect to the hydrazone **HZ** in the beginning of reaction indicates that hemiaminal **H2** is formed by low energy demanding addition reaction from the hydrazone **HZ** corresponding with Path 1. Unexpectedly, bis-hemiaminal **H3** shows relatively high half-life and is formed in a high amount in the reaction mixture. After the initial burst effect, the reaction mode switch to Path 1 with the rate-determining step to be the dehydration of the hemiaminal **H2** to form the product **2**. Kinetic profile for the reaction of **Pz** with hydrazine is shown in Fig. 3B. The evolution of reaction intermediates follows similar trends as for the pyridinyl case, however, with some modulations. For example, bis-hemiaminal **H3** is formed only in minor extent. Then, the hemiaminal **H2** shows relatively higher abundance mostly due to reduced formation of the hemiaminal **H3** which more easily (with respect to the pyridinyl hemiaminal) reverts back to reactants via low energy demanding addition steps of Path 2. In the course of reaction surprisingly stable hemiaminal **H2** and product **1** gradually precipitate from the solution as was confirmed by vibrational spectroscopy and X-ray analysis, respectively (see the SI), and this partially affects kinetic behavior. In the solid state, the hemiaminal **H2** is stable compound which slowly (within days) dehydrates to the azine **1** changing color from white to yellow. We also tried to monitor the kinetic progress in methanol, however, the situation was complicated due to the aldehyde-hemiacetal pre-equilibrium (see Fig. S11). Moreover, the presence of rotamers of the hydrazone **HZ** further complicates the proper integration of spectra, hence it was difficult to obtain the reliable kinetic parameters.

**Figure 4:**
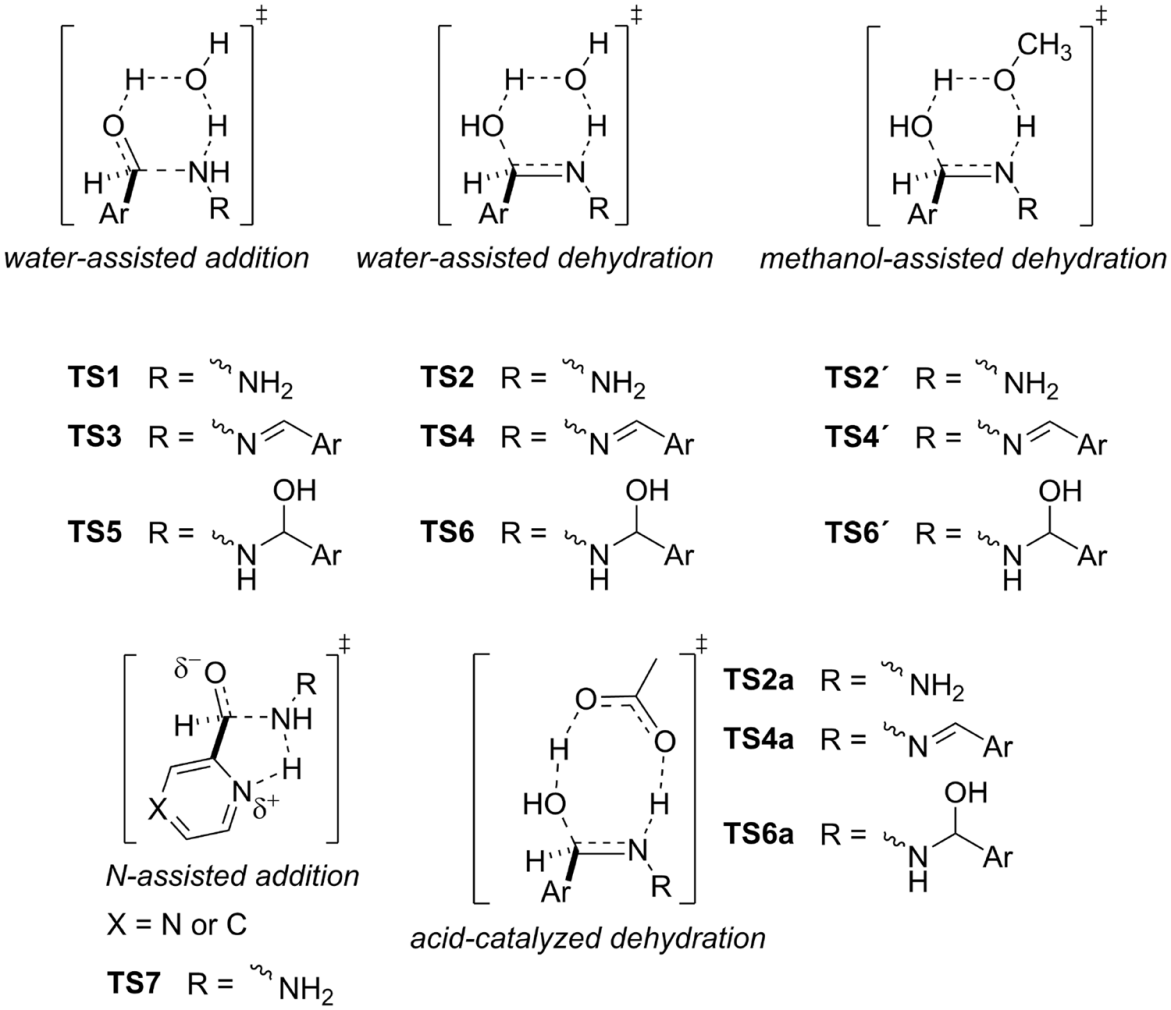
Schematic representation of the transition states exhibited throughout the azine formation. Ar stands for pyrazine or pyridine ring.

Reaction mechanisms for pyrazinyl and pyridinyl azines (**1** and **2**) formations have been investigated by density functional theory calculations in order to shed light on experimental results. All optimized structures in the gas-phase employed here are gathered in the SI2. First, nucleophilic addition and dehydration steps were investigated regarding acceleration by a water molecule (contained in solvent or generated as a byproduct of azomethine formation) acting as a catalyst by forming a pseudo-six-membered rings in transition state structures, see Fig. 4. As another common solvent for synthesis of azomethine compounds is methanol we have performed key calculations in the bulk methanol in addition to the calculations in the bulk THF. Here, it can be generally expected that an active catalytic water molecule involved in transition states is replaced by a methanol molecule. The respective transition states are marked with apostrophe. We have also proposed another possible pathway for the first addition step, i.e. five-membered transition of the type **TS7**, as is depicted in Fig. 4. Here, the proton transfer from the hydrazine to the nitrogen atom of aromatic ring is facilitated via geometrically feasible pseudo-five-membered ring while nitrogen-carbon bond is formed in a concerted process. Pliego and Rufino have recently demonstrated a strong catalytic effect of traces of acidic impurities in the formation of aza-Micheal products^47^. Since acetic acid was used in the preparation of the reactants **Py** and **Pz** and once is difficult to completely eliminate the impurities we investigated acetic acid catalyzed mechanism via eight-membered transition state for the dehydration steps. The respective transition states are marked with **a**. The importance of the catalysis is dispatched in Fig. S2 were the dehydration step from **H1** to **HZ** with nude reactants reaches energy barriers well above 200 kJ/mol both in the gas-phase and bulk solvents and hence this non-assisted process without any catalytic effect cannot occur spontaneously under ambient conditions. This is essentially due to high ring strain of pseudo-4-memebered ring involved in **TS**_**na**_.

### Water and methanol-assisted mechanisms

**Figure 5:**
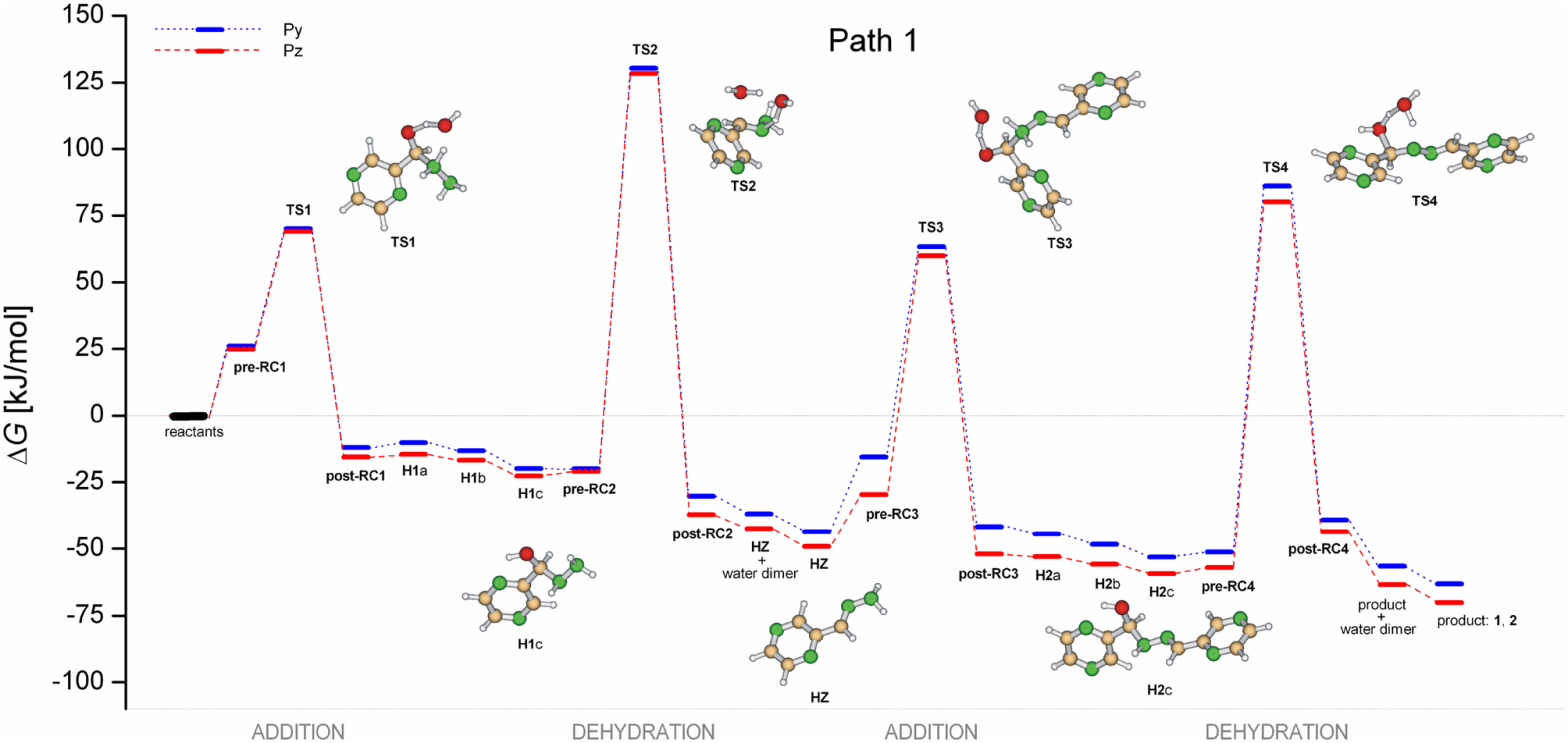
Reaction free energy profiles in the gas-phase for pyrazinyl and pyridinyl azines formation catalyzed by water via the sequence addition-dehydration-addition-dehydration. Selected pyrazinyl intermediates are shown.

**Figure 6:**
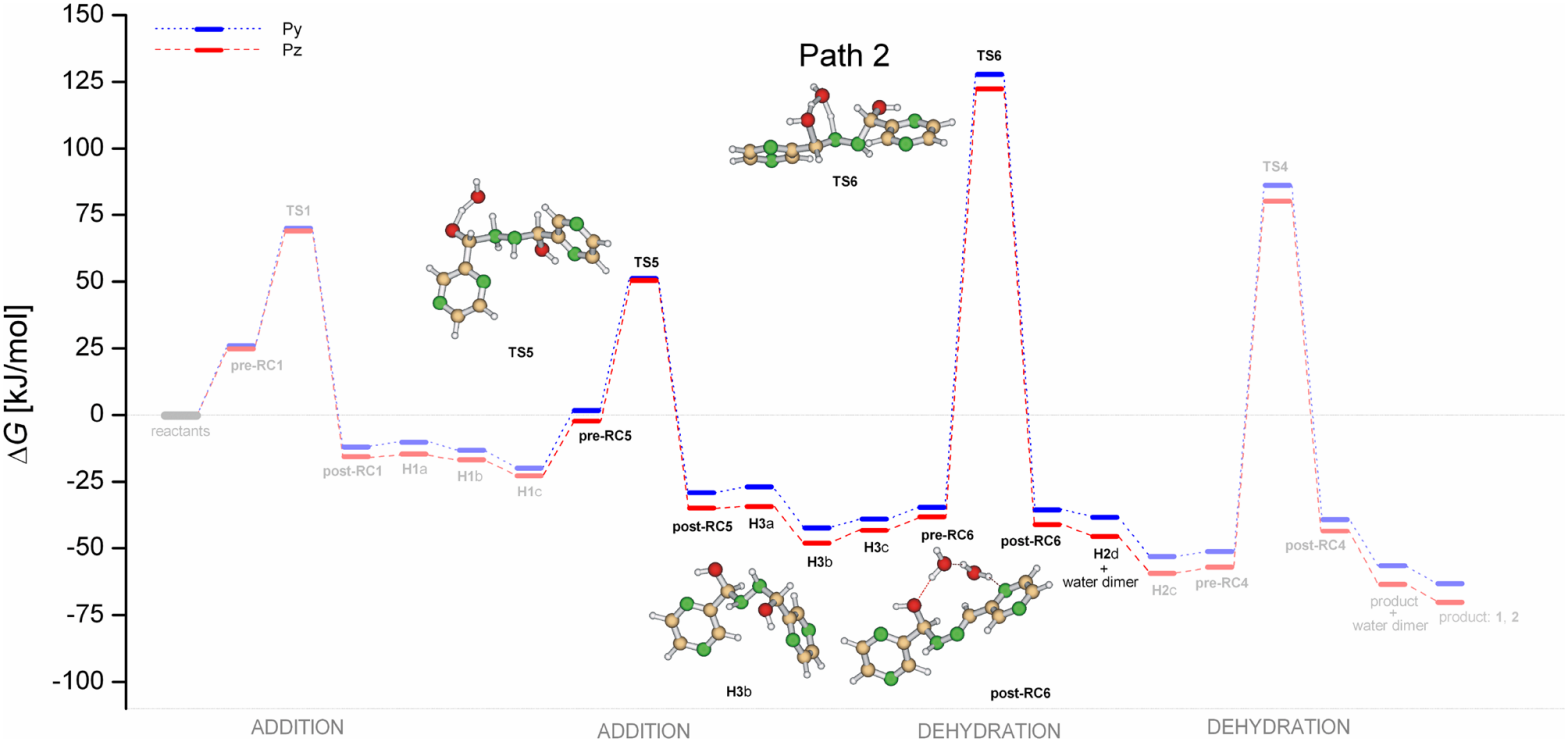
Reaction free energy profiles in the gas-phase for pyrazinyl and pyridinyl azines formation catalyzed by water via the sequence addition-addition-dehydration-dehydration. Selected pyrazinyl intermediates are shown.

The addition of hydrazine to the carbaldehyde group in the first step results in the hemiaminal **H1** (after decomplexation of the post-reactive complex **post-RC1**) via weakly bound pre-reactive complex **pre-RC1** and transition state **TS1**, see Figs. 5 and 6. Along the Path 1, Fig. 5, (sequence addition-dehydration-addition-dehydration), hemiaminal **H1** undergoes two consecutive isomerization steps, forming the most stable isomer **H1**c. Isomerization barriers which are not shown are significantly lower than activation energies. Also, we have found that during isomerization process inversion of configuration on nitrogen atom occurs as a barrierless process. Here, anomeric effect (hyperconjugation) is considered source of the enforced conformation with the lowest energy. Moreover, the hydrogen of hydroxyl group is pointed toward the nitrogen atom of aromatic ring providing additional stabilization. In the following step, the hemiaminal **H1** weakly bounds with a water molecule forming **pre-RC2** and dehydrates via **TS2** to form hydrazone **HZ** after releasing water-dimer (one assisting and one releasing water molecule). In the second addition step, the hydrazone **HZ** react with the second carbaldehyde to form hemiaminal **H2** via transition state **TS3**. Finally, hemiaminal **H2** dehydrates to form product (**1**, **2**). In general, free activation energies of dehydration are significantly higher than the addition ones, though the second dehydration barrier is lower than the first one as it leads to product with extended conjugation. The first addition barrier is decreased with respect to the second one as the hydrazone **HZ** is less nucleophilic than hydrazine. The overall reaction is exoergic by about 70 kJ/mol for pyrazine and 63 kJ/mol for pyridine carbaldehyde.

Along the Path 2 (sequence addition-addition-dehydration-dehydration) as shown in Fig. 6, hydrazine undergoes two consecutive addition steps forming the hemiaminal **H3** via transition states **TS1** and **TS5**. After isomerization steps (from **H3**a to **H3**c) and formation of weakly bound water-association complex **pre-RC6** the hemiaminal **H3** dehydrates to form hemiaminal **H2** via the transition state **TS6** and the cyclic trimer **post-RC6**. This process is connected with the highest activation energy within both free energy surfaces. The last dehydration step is the same as for the Path1.

**Figure 7:**
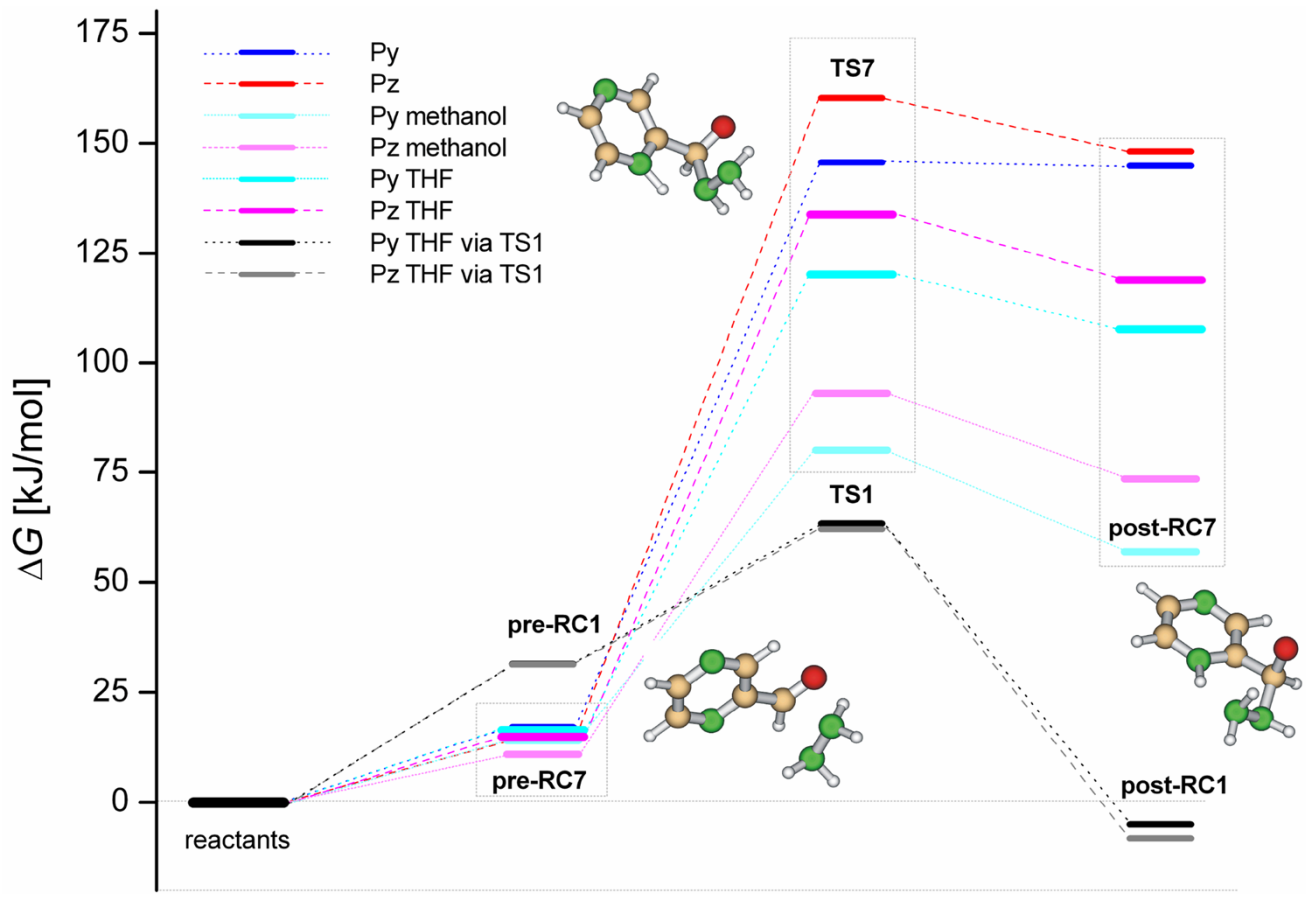
Reaction free energy profiles for water and nitrogen-assisted addition of hydrazine to pyrazine and pyridinecarbaldehydes in the gas-phase and bulk solvents. Selected pyrazinyl intermediates of nitrogen-assisted addition are shown.

Consideration of the solvent effect has been done using the SMD continuum solvent model. Results of calculations for the first addition step which is common for both mechanistic paths are shown in Fig. 7. Water-assisted addition of hydrazine to **Py** and **Pz** shows the barriers slightly above 60 kJ/mol, that is fast reaction. Nitrogen-assisted addition via transition state **TS7** leading to zwitter-ionic intermediate **post-RC7** shows significantly higher activation energies in the gas-phase. However, polar bulk solvent stabilizes **TS7** and zwitter-ionic complex **post-RC7** as these intermediates exhibit partially separated charges. Nevertheless, the energy barriers of 120.2 kJ/mol (Py) and 133.6 kJ/mol (Pz) for this process are significantly higher with respect to the water-assisted addition and therefore the nucleophilic addition with intramolecular assisted proton transfer is less probable for the studied reaction. However, the relative energy levels of intermediates of ionic mechanism are closer to water-assisted ones when more polar solvent such as methanol is considered. This leads to energy barriers of 80.1 kJ/mol (Py) and 93.1 kJ/mol (Pz) and it shows that in more polar solvents as simulated by the SMD model the ionic pathway may be regarded as a competitive mechanism for the formation of hemiaminals. Noteworthy, the opposite trend in stability of intermediates of pyrazine *versus* pyridine is observed in the case of ionic mechanism, manifesting the higher basicity of the pyridine with respect to the pyrazine ring.

**Figure 8:**
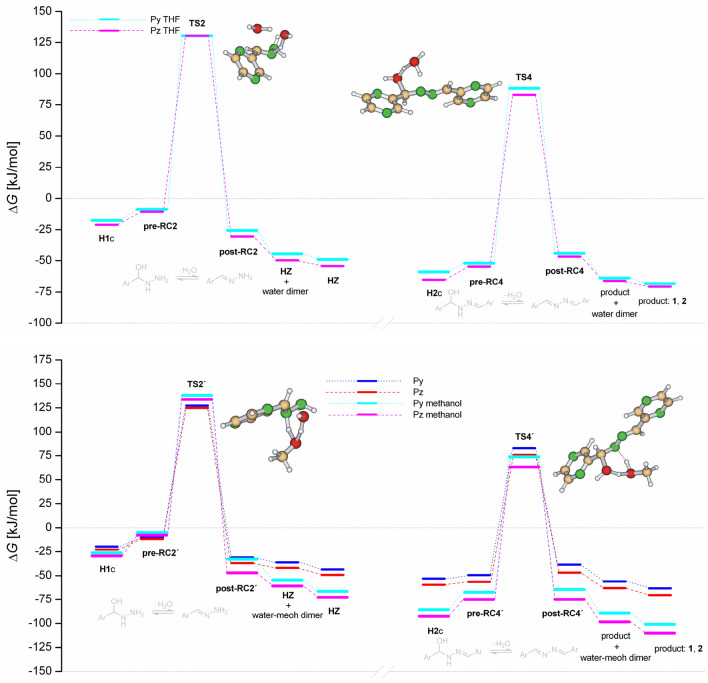
Reaction free energy profiles for water and methanol-assisted dehydrations of pyridinyl and pyrazinyl hemiaminals **H1**c and **H2**c in the bulk THF (top) and bulk methanol (bottom). Pyrazinyl transition states are shown.

**Figure 9:**
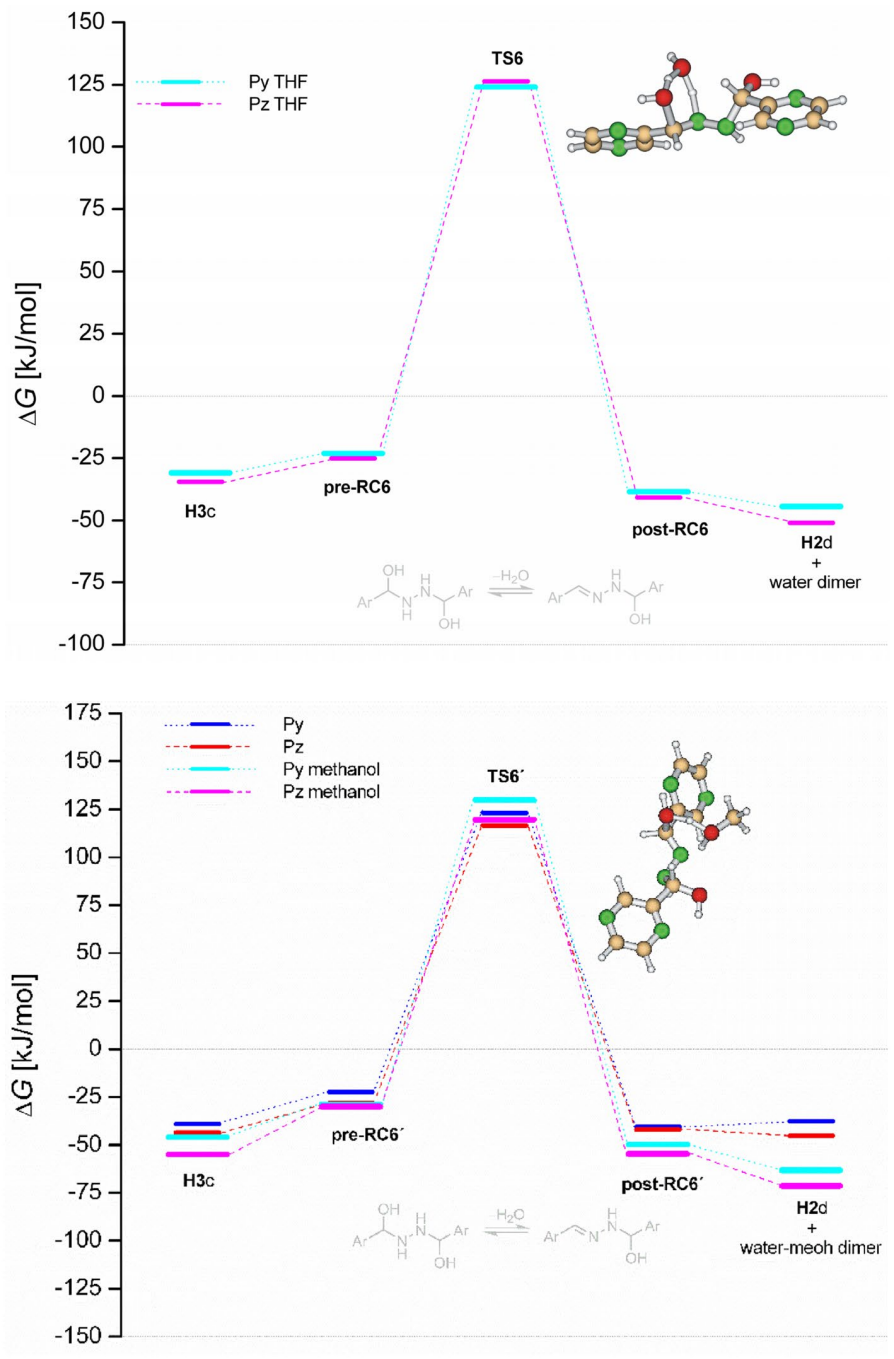
Reaction free energy profiles for water and methanol-assisted dehydration of pyridinyl and pyrazinyl hemiaminal **H3**c in the bulk THF (top) and bulk methanol (bottom). Pyrazinyl transition states are shown.

Calculations in the bulk THF predict the nearly similar activation barriers for dehydration steps in Path 1 as is illustrated by Fig. 8 (top). The similar picture provided calculations in the bulk methanol, Fig. 8 (bottom). However, the free activation barriers of about 150 kJ/mol in THF and 160 kJ/mol in methanol are high considering that the reactions proceed at ambient conditions. Moreover, the theoretical results in THF are inconsistent in relation to the experimental observation, where the rate-determining step (dehydration of **H2** forming product) proceeds with the barriers slightly below 100 kJ/mol (see Table 2). Even higher barriers of about 160 kJ/mol for water-assisted process and 175 kJ/mol for methanol-assisted process are predicted for dehydration in Path 2 (from **H3** to **TS5**), see Fig. 9.

**Table 2:** Theoretical (DFT) and experimental (EXP) Gibbs free activation energies for dehydration of the hemiaminal **H1**c (Δ*G*^‡^_2a_), **H2**c (Δ*G*^‡^_4a_) and **H3**c (Δ*G*^‡^_4a_).

Reactant	DFT			EXP
	Δ*G*^‡^_2a_	Δ*G*^‡^_6a_	Δ*G*^‡^_4a_	
Pz	93.7	110.4	102.5	98.7
Py	91.0	107.6	98.6	95.9

### Acid catalyzed mechanism

**Figure 10:**
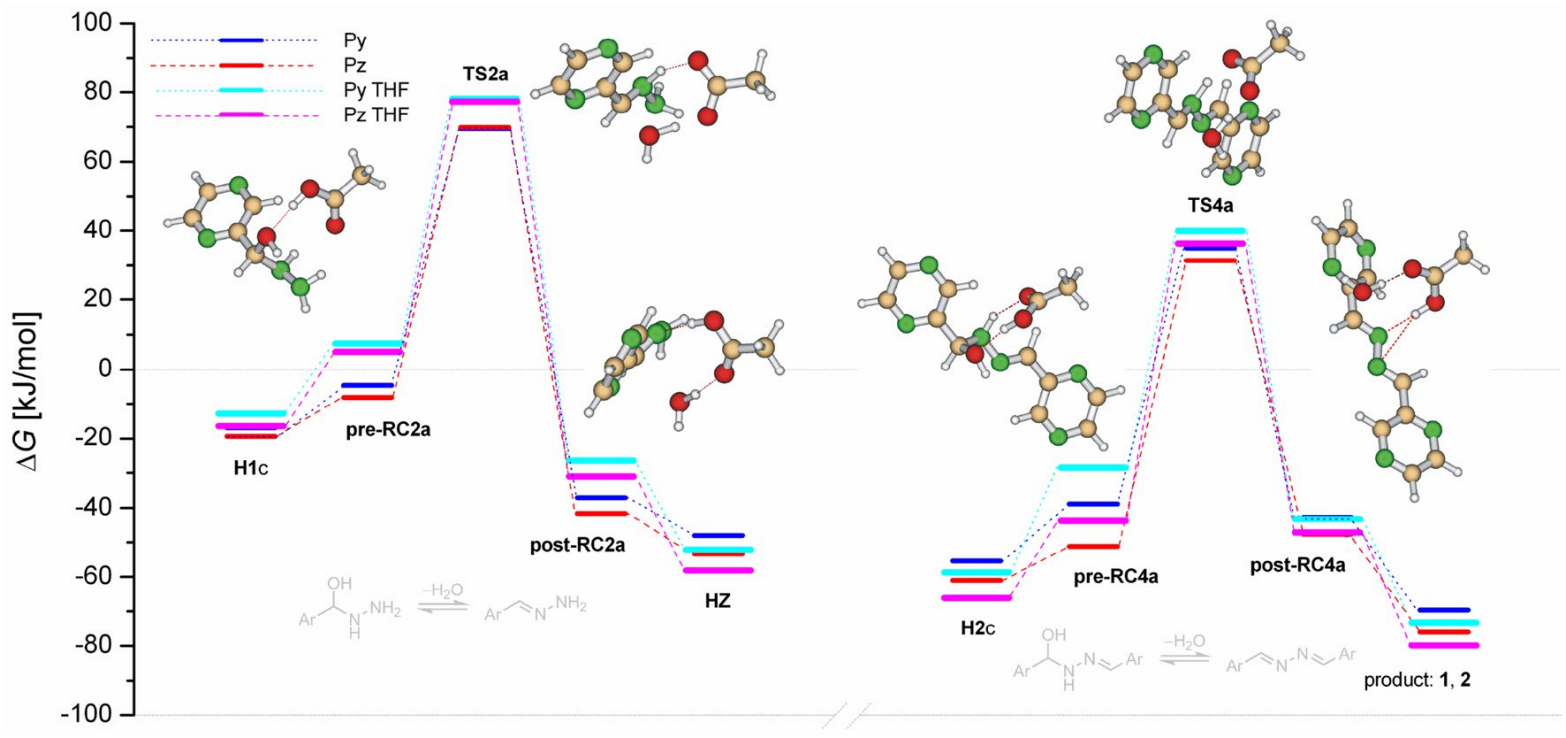
Reaction free energy profiles for acetic acid-assisted dehydration of pyridinyl and pyrazinyl hemiaminal **H1**c and **H2**c in the gas-phase and bulk THF obtained at SMD/M06-2X/def2-TZVPPD level. Selected pyrazinyl intermediates are shown.

**Figure 11:**
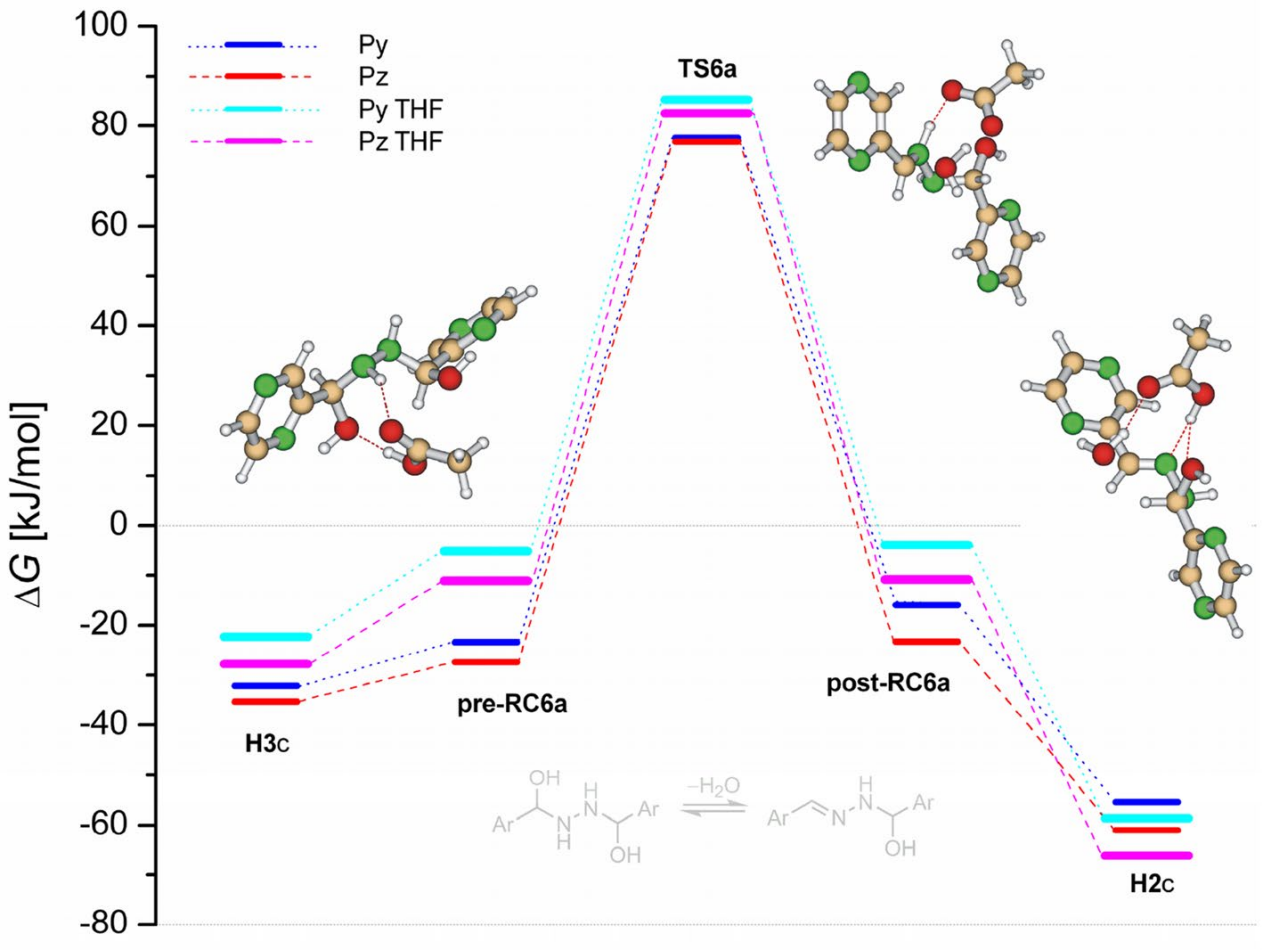
Reaction free energy profiles for acetic acid-assisted dehydration of pyridinyl and pyrazinyl hemiaminal **H3**c in the gas-phase and bulk THF obtained at SMD/M06-2X/def2-TZVPPD level. Selected pyrazinyl intermediates are shown.

The preceding sections imply that the theoretical investigation gave reasonable mechanistic insight into azine formation where water or methanol molecules can account sufficiently for H-transfer process in the addition steps. However, the significantly higher energy barriers for dehydration steps with respect to the experimental observation indicate that another mechanistic path must be found. As the reactants **Py** and **Pz** are prepared using acetic acid workup and once is difficult to completely eliminate traces of impurities acetic acid-catalyzed mechanism was considered here. Although the presence of acetic acid is below the detection limit of ordinary techniques Pliego and Rufino^47^ theoretically proposed that aza-Micheal product may be effectively formed even when trace and undetectable amount of acid originated from reagents or solvent is present in a reaction mixture using similar level of theory. As they also showed that an amine molecule has a low catalytic effect the amine-catalyzed mechanism was excluded here. Also, the importance of acid catalysis was recently demonstrated by Halasz et al.^48^ in mechanochemical formation of imine derived from *p*-nitrobenzaldehyde and *p*-nitroaniline. Results on acetic acid-catalyzed azine formations are shown in Figs. 10 and 11. The change of active catalytic molecule from water or methanol to acetic acid leads to significant reduction of free energy barriers from approx. 150–160 kJ/mol (Fig. 8) to 91–94 kJ/mol for the first dehydration step to 99–103 kJ/mol for the second dehydration (rate-determining) step, see Table 2. As can be seen from enthalpy profiles (Figs. S3 and S4), complexation of hemiaminals with acetic acid provides significant stabilization. Similar observations were reported in the investigations of the first steps of the Strecker synthesis^36^ or in the dimer formation between common oxygen-containing functional groups^49^. However, considering the pre-reaction complexes as reference point for energy barrier calculation is erratic as was discussed in the literature^50^. In agreement with experiments, calculations correctly predict the rate-determining step to be dehydration of the hemiaminals **H2** forming products. Also, dehydration of the hemiaminal **H3** (Fig. 11) is the highest energy demanding step, in accordance with experimental observations where the hemiaminal **H3** proceeds back to reactants. Significantly, the results of acid-catalyzed mechanism for the rate-determining step (ΔG^‡^_4a_) of azines formation show excellent agreement with experimental values with the difference of about 3–4 kJ/mol only, well below an uncertainty of the M06-2X functional for barrier height (2.6 kcal/mol)^51^. Based on this picture, we can postulate that acetic acid-catalyzed mechanism via synchronous eight-membered transition state, where acetic acid works as bifunctional catalyst brings the theory and experiment into very good agreement.

## Conclusion

In summary, combined experimental and theoretical studies have been used to explore possible pathways for the formation of bis-azomethine compounds. It has been shown that order of elementary steps follows the sequence addition-dehydration-addition-dehydration which is preferred over the sequence addition-addition-dehydration-dehydration. Despite the fact that unstabilized hemiaminals have previously been observed mostly under specialized conditions, we were able to clearly observe bis-hemiaminal in NMR spectra without using any specific methods. Moreover, pyrazinyl hemiaminal was successfully isolated from reaction mixture as an amorphous material and characterized by vibrational spectroscopy. The importance of active catalysis has been shown as the catalytic molecules are involved in the relaxed transition states which results in the significant free energy reduction with respect to the direct four-membered transition states. Acetic acid-catalyzed mechanism via synchronous eight-membered transition state brings the theory and experiment into very close agreement. The results described here should provide opportunities in exploration, designing and analysis of other more complex hemiaminal and azomethine-based system, especially dynamic polymers.

### Supplementary Information

Supplementary Information 1.

Supplementary Information 2.

## Data Availability

All data generated or analyzed during this study are included in this published article and its supplementary information files.
