# Supplementary material for: Pyrazinyl and pyridinyl bis-azomethines formation: an experimental and computational study
Source: Sci Rep. 2023 Oct 13;13:17383. doi: 10.1038/s41598-023-44585-7 (PMC10575867; doi:10.1038/s41598-023-44585-7)
Supplement: Supplementary file 1 — Supplementary Information 1. [file 41598_2023_44585_MOESM1_ESM.docx]

**Pyrazinyl and Pyridinyl Bis-Azomethines Formation: An Experimental Computational Study**

Radek Coufal^1*^ and Jiří Vohlídal^2^

*^1^Department of Science and Research, Faculty of Health Studies, Technical University of Liberec, Studentská 1402/2, 461 17 Liberec 1, Czech Republic, ^2^Department of Physical and Macromolecular Chemistry, Faculty of Science, Charles University, Hlavova 8/2030, 128 40 Prague 2, Czech Republic*

**Supporting Information**

TABLE OF CONTENTS

**General**	2

**Synthesis**	2

**Kinetic data**	4

**Computational data**	5

**X-ray structure data**	7

**^1^H NMR and ^13^C NMR spectra**	8

**IR and Raman spectra**	14

**References**	16

**General.** NMR spectra were recorded on Bruker AVANCE-III 600 and VNMRS300 spectrometers. ^1^H NMR and spectra are referenced to residual solvent signal. High-resolution mass spectra were obtained with LTQ Orbitrap XL (Thermo Fisher Scientific) spectrometer. IR spectra were recorded on Nicolet Avatar 370 FT-IR using KBr-diluted samples or SiC (400 GRIT SI-CARB PAPER) and diffuse reflectance technique (DRIFT). Raman spectra were obtained with DXR Raman microscope (Thermo Fisher Scientific) in solid state using 532, 633 or 780 nm excitation (1mW) Crystallographic data were collected on Nonius Kappa CCD diffractometer equipped with Bruker Apex-II CCD detector and a Cryostream Cooler (Oxford Instruments) at 150(2) using graphite-monochromated Mo K*α* radiation (*λ* = 0.71073 Å). The crystal structure was solved by direct methods (SHELXS),^1^ and the structure model was refined by full matrix least squares based on *F*^2^ (SHELXL2018).^2^ The non-hydrogen atoms were refined with anisotropic displacement parameters. The hydrogen atoms residing on the oxygen atoms were identified on the difference electron density maps and refined as riding atoms with *U*_iso_(H) set to 1.2-times *U*_eq_(O). Hydrogen atoms residing on the carbon atoms were included in their theoretical positions and refined analogously. Geometric data as well as structural drawings were obtained with a recent version of the PLATON progam.^3^ Tetrahydrofuran (THF) was distilled from sodium/benzophenone ketyl under argon atmosphere. Methanol was distilled from dry CaO under argon atmosphere Solvents for workup and chromatography and chemicals obtained from commercial suppliers were used without any further purification.

**Synthesis.** Pyrazine-2-carbaldehyde (**Pz**) was prepared by the reaction sequence shown in Scheme S1.

**Scheme S1** Synthesis of pyrazine-2-carbaldehyde.

**Methyl pyrazinoate.** This compound was prepared by a modification of the procedures of Goodson and Bark.^4^ 2-Pyrazine carboxylic acid (25.70 g; 207 mmol) was suspended in dry MeOH (250 mL) and concentrated H_2_SO_4_ (ca. 10 drops) was added. The mixture was heated to reflux for 2 days. After cooling to ambient temperature, the solution was treated with solid NaHCO_3_ (5 g) for 16 h. The mixture was concentrated under reduced pressure, filtered, and the residue was diluted with DCM. After drying over MgSO_4_, the solution was filtered through alumina column. The solvent was removed under reduced pressure, and the residue was sublimed in vacuo (ca. 110 °C) to give 20.9 g (73 %) of the title compound as a white crystalline solid. ^1^H NMR (299.94 MHz, CDCl_3_): *δ* = 9.34 (d, 1H, pz, ^4^*J* = 1.5 Hz), 8.80 (d, 1H, ^3^*J* = 2.4 Hz), 8.74 (dd, 1H, ^3^*J* = 2.4 Hz, ^4^*J* = 1.5 Hz), 4.06 (s, 3H, CH_3_) ppm, in agreement with the spectrum published in the literature.^4^

**Pyrazine-2-carbaldehyde (Pz).** This compound was prepared by a procedure of Ligier *et al*.^5^ To a THF (20 mL) solution of methylpyrazinoate (0.94 g; 6.9 mmol), at −78 ºC, a diethylether solution of LiAlH_4_ (3 mL; 6 mmol) was slowly added over a period of 1h and then stirred at this temperature for another 1h. The reaction was quenched by the slow addition of acetic acid (0.98 mL; 17 mmol). The solvent was removed under reduced pressure and the residue dissolved in 7.5 mL of aqueous solution of HCl (2.5 mol L^−1^) and extracted with chloroform (4 × 8mL). The organic layer was washed with saturated NaHCO_3_ (1 × 10 mL), and dried over anhydrous MgSO_4_. After filtration, the solvent was removed under reduced pressure. Column chromatography (silicagel, hexane/ethyl acetate 1/1) furnished pure aldehyde as a pale yellow oil (30 mg; 4 %). ^1^H NMR (299.94 MHz, CDCl_3_): *δ* = 10.12 (s, 1H), 9.33 (d, 1H), 8.78 (d, 1H), 8.73 (t, 1H) ppm, in agreement with the spectrum published in the literature.^5^

**Azine 2**. This compound was prepared by a slightly modified procedure of Kennedy *et al*.^6^ To a solution of pyrazine-2-carbaldehyde (1.9 mL; 20 mmol) in methanol, a solution of hydrazine hydrate (0.5 mL; 10 mmol) was added at l. t. After 30 min the precipitate was collected by filtration, washed with methanol and dried to afford the title compound as yellow powder (227 mg, 11 %). ^1^H NMR (299.94 MHz, (CD_3_)_2_SO): *δ* = 8.71 (d, *J* = 3.0 Hz, 2H), 8.56 (s, 2H), 8.12 (d, *J* = 6.0 Hz, 2H), 7.96 (t, *J* = 9.0 Hz, 6.0 Hz, 2H), 7.54 (t, *J* = 6.0 Hz, 9.0 Hz, 2H) ppm, in agreement with the spectrum published in the literature.^6^

**Pyrazinyl hemiaminal H2.** A mixture of pyrazine-2-carbaldehyde (60 mg, 0.56 mmol) in THF and hydrazine in 1M THF (0.28 mL; 0.28 mmol) was stirred at r. t. for 17 h. The resultant precipitate was filtered off, washed with ethanol, and shortly dried in vacuo to afford 10.6 mg of white powder. Noteworthy, reaction can be performed in methanol or ethanol with very similar reaction progress. HRMS (ESI): calc. for C10H10ON6Na 253.08083, found 253.08093. NMR, IR and Raman spectra are given below.

**Azine 1.** As per the procedure for the pyrazinyl hemiaminal **H2**, reaction time 72 h., to afford **1** as yellow to brownish crystalline solid (33 mg, 28 %). HRMS (ESI): calc. for C10H8N6Na 235.07026, found 235.07027. NMR, IR and Raman spectra are given below.

**Kinetic data**

**Figure S1**. Kinetic build-up curves for the rate-determining steps of azine **2** (A) and azine **1** (B) formations fitted with second order polynomial.

**Computational data**

**Figure S2** Reaction free energy profiles for dehydration of pyridinyl and pyrazinyl hemiaminal **H1**c with no catalysis in the gas-phase, bulk THF and bulk methanol. Selected pyrazinyl intermediates are shown.

**Figure S3** Reaction enthalpy profiles for acetic acid-assisted dehydration of pyridinyl and pyrazinyl hemiaminal **H1**c and **H2**c obtained at M06-2X/def2-TZVPPD level.

**Figure S4** Reaction enthalpy profiles for acetic acid-assisted dehydration of pyridinyl and pyrazinyl hemiaminal **H3**c obtained at M06-2X/def2-TZVPPD level.

**Figure S5** Comparison of theoretical (red) and experimental structures for azines **1** (left) and **2** (right). The experimental structure of azine **2** was reported in ref. [7].

**X-ray structure data**

**Figure S6** X-ray structure of the azine **1**.

**Table S1.** Crystallographic data

| Compound | **1** |
| --- | --- |
| Formula | C_10_H_8_N_6_ |
| *M* | 212.22 |
| Crystal size (mm^3^) | 0.53 × 0.08 × 0.03 |
| System | Triclinic |
| Space Group | *P*¯1 (No. 2) |
| *a*/Å | 3.7149 (2) |
| *b*/Å | 5.0895 (3) |
| *c*/Å | 13.4257 (9) |
| α/° | 96.611 (3) |
| β/° | 94.220 (2) |
| γ/° | 99.040 (2) |
| *V*/Å^3^ | 247.93 (3) |
| *Z* | 1 |
| D_c_/g cm^–3^ | 1.421 |
| T/K | 120 |
| μ/mm^–1^ | 0.10 |
| *T*_min_, *T*_max_ | 0.94, 1.00 |
| θ_min_–θ_max_/° | 3.1–27.5 |
| Measured refl. | 4560 |
| Unique refl. | 1131 |
| *R*_in_*_t_^a^* | 0.021 |
| Observed Refl.*^b^* | 1021 |
| *R* [*F*^2^> 2σ(*F*^2^)]*^c^* | 0.038 |
| *wR*(*F*^2^)*^d^* | 0.100 |
| *S^e^* | 1.11 |
| Δρ_max_; Δρ_min_/e Å^–3^ | 0.32; -0.21 |
| CCDC deposition number | 2253081 |

*^a^ R*_int_ = Σ⏐*F*_o_^2^ - *F*_o,mean_^2^⏐/Σ*F*_o_^2^, *^b^ I*>2δ(*I*),*^c^* *R*(*F*) = Σ⏐⏐*F*_o_⏐ - ⏐*F*_c_⏐⏐/Σ⏐*F*_o_⏐, *^d^wR*(*F*^2^) = [Σ(*w*(*F*_o_^2^ - *F*_c_^2^)^2^)/(Σ*w*(*F*_o_^2^)^2^)]^½^,*^e^S*= [Σ(*w*(*F*_o_^2^ - *F*_c_^2^)^2^)/(*N*_diffrs_ - *N*_params_)]^½^.

**^1^H NMR and ^13^C NMR spectra**

**Figure S7** Example of the time-dependent ^1^H NMR spectra for the reaction of pyridine-2-carbaldehyde with hydrazine in THF-d8. A) *t* = 9 min; B) *t* = 547 min; C) *t* = 1267 min. Peaks used for integration are marked.

**Figure S8** ^1^H NMR spectra for the reaction of pyridine-2-carbaldehyde with hydrazine in THF-d8 with signal assignment. Reaction time 9 min (top) and 547 min (bottom).

**Figure S9** Example of the time-dependent ^1^H NMR spectra for the reaction of pyrazine-2-carbaldehyde with hydrazine in THF-d8. A) *t* = 8 min; B) *t* = 544 min; C) *t* = 1358 min. Peaks used for integration are marked.

 **Figure S10** ^1^H NMR spectra for the reaction of pyrazine-2-carbaldehyde with hydrazine in THF-d8 with signal assignment. Reaction time 8 min (top) and 544 min (bottom).

**Figure S11** Example of the time-dependent ^1^H NMR spectra for the reaction of pyridine-2-carbaldehyde with hydrazine in MeOD-d3. A) *t* = 5 min; B) *t* = 86 min; C) *t* = 353 min. Rotamers of the hydrazone **HZ** are marked as **HZ (A)** and **HZ (B)**. In this case **R** stands for pyridinyl hemiacetal.

**Figure S12** ^1^H NMR spectrum of pyrazinyl hemiaminal **H2** in DMSO-d6.

**Figure S13** ^13^C NMR spectrum of pyrazinyl hemiaminal **H2** in DMSO. Peaks (asterix) of the azine **1** are visible due to the longer standing in NMR solvent during fid accumulation.

**Figure S14** ^1^H NMR spectrum of the azine **1** in DMSO-d6.

**Figure S15** ^13^C NMR spectrum of the azine **1** in DMSO-d6.

**IR and Raman spectra**

**Figure S16** IR spectrum of pyrazinyl hemiaminal **H2**.

**Figure S17** IR spectrum of the azine **1**.

**Figure S18** Raman spectra of pyrazinyl hemiaminal **H2** and the azine **1**.

**References**

[1] Sheldrick, G. M. *Acta Crystallogr. Sect. A: Found. Crystollogr.***2008**, *64*, 112.

[2] SHELXL: Sheldrick, G. M. *Acta Cryst.***2015**, *C71*, 3.

[3] Spek, A. L. *Acta Cryst.* **2009**, *D65*, 148.

[4] (a) Goodson, P. A.; Oki, A. R.; Glerup, J.; Hodgson, D. J. *J. Am. Chem. Soc.* **1990**, *112*, 6248. (b) Bark, T., PhD thesis, Universität Freiburg (Schweiz), 2004.

[5] Ligier, C. B. P.; Vinsentin, L. C.; Giacomini, R.; Filgueiras, C. A. L.; Miranda, P. M. L. *Tetrahedron Lett*. **2009**, 50, 4030.

[6] Kennedy, A. R.; Brown, K. G.; Graham, D.; Kirkhouse, J. B.; Kittner, M.; Major, C.; McHugh, C. J.; Murdoch, P.; Smith, W. E. *New J. Chem*. **2005**, 29, 826.

[7] Kesslen, E. C.; Euler, W. B. *Chem. Matter.*, **1999**, *11*, 336-340.
